# The protection motivation theory for predict intention of COVID-19 vaccination in Iran: a structural equation modeling approach

**DOI:** 10.1186/s12889-021-11134-8

**Published:** 2021-06-17

**Authors:** Alireza Ansari-Moghaddam, Maryam Seraji, Zahra Sharafi, Mahdi Mohammadi, Hassan Okati-Aliabad

**Affiliations:** Health Promotion Research Center, Zahedan University of Medical Sciences, Zahedan, Iran

**Keywords:** COVID-19, Vaccination, Intention, Structural equation modeling, Iran

## Abstract

**Background:**

Many efforts are being made around the world to discover the vaccine against COVID-19. After discovering the vaccine, its acceptance by individuals is a fundamental issue for disease control. This study aimed to examine COVID-19 vaccination intention determinants based on the protection motivation theory (PMT).

**Methods:**

We conducted a cross-sectional study in the Iranian adult population and surveyed 256 study participants from the first to the 30th of June 2020 with a web-based self-administered questionnaire. We used Structural Equation Modeling (SEM) to investigate the interrelationship between COVID-19 vaccination intention and perceived susceptibility, perceived severity, perceived self-efficacy, and perceived response efficacy.

**Results:**

SEM showed that perceived severity to COVID-19 (β = .17, *p* < .001), perceived self-efficacy about receiving the COVID-19 vaccine (β = .26, *p* < .001), and the perceived response efficacy of the COVID-19 vaccine (β = .70, *p* < .001) were significant predictors of vaccination intention. PMT accounted for 61.5% of the variance in intention to COVID-19 vaccination, and perceived response efficacy was the strongest predictor of COVID-19 vaccination intention.

**Conclusions:**

This study found the PMT constructs are useful in predicting COVID-19 vaccination intention. Programs designed to increase the vaccination rate after discovering the COVID-19 vaccine can include interventions on the severity of the COVID-19, the self-efficacy of individuals receiving the vaccine, and the effectiveness of the vaccine in preventing infection.

## Background

Vaccines are one of the cost-effective measures of prevention [1]. Immunization against infectious diseases annually prevents millions of deaths by affecting the immune system [2]. The spread of COVID-19 as an emerging disease in the world requires immediate action, including the production of vaccines, which can be an effective measure to protect people against this disease [3]. Many efforts are being to prevent individuals from getting COVID-19 through vaccination [4]. After providing the vaccine, the critical issue is its acceptance by the individuals. A survey of American adults found that about a third of them will accept COVID-19 vaccination [5]. Also, A report from the Centers for Disease Control and Prevention found that less than half of American adults vaccinated against the flu in the 2018–2019 season [6].

Evidence shows that the rate of influenza vaccination is low in Asian populations [7], and this rate in Iran is much lower than expected by the World Health Organization [8]; however, Iran is one of the countries that announced the highest agreement on the importance of the vaccine [9]. The evidence shows that misconceptions are among the main reasons for not getting the flu vaccine [10].

According to a global report in 2017, most countries report that people are hesitant about vaccination [11]. Factors affecting COVID-19 vaccination acceptance may be as important as the discovery of the vaccine [12]. It is unclear how effective the pandemic status is in accepting the COVID-19 vaccine, and doubts about the vaccine acceptance remain [13]. Policymakers can identify factors related to vaccine acceptance to guide effective interventions to increase vaccination acceptance in the population [14]. The theory of protection motivation (PMT) is one of the most recognized expectancy-value theories that explain the effects of fear appeals on attitude change [15]. Behavioral change interventions widely use fear appeal to be effective. Fear appeals when messages contain a description of perceived susceptibility, perceived severity, and expressions of response efficacy can positively affect individuals’ knowledge, attitude, and performance, especially in onetime behaviors (e.g., Covid-19 vaccination) [16, 17].

A recent study examining the effectiveness of the PMT in predicting seasonal influenza vaccination intent has shown that this model is a good predictor [18]. Also, a survey that used protective motivation theory to predict COVID-19 preventive behaviors in Iran showed that the response efficacy and self-efficacy predicted COVID-19 protective behaviors [19]. Furthermore, evidence shows that threat and coping appraisal in hospital staff were predictors of protection motivation during the COVID-19 pandemic [20]. To the best of our knowledge, no studies have so far examined the predictors of intention to vaccinate COVID-19 using the PMT. This study aimed to investigate the predictors of COVID-19 vaccination intention using the PMT in the Iranian population.

## Methods

### Study design

We conducted a cross-sectional study in the Iranian adult population 18 years and older and surveyed 265 participants from the first to the 30th of June 2020 with a web-based self-administered questionnaire. We made a questionnaire based on the conceptual framework of the PMT on the Porsline, an online survey platform in Iran (https://survey.porsline.ir). We recruited participants with the self-selection sampling method and posted the online survey link on Telegram and WhatsApp, two of Iran’s most widely used social media platforms. The questionnaire began with an information letter about the study’s purpose, how to answer questions, and informed consent to participate in the study.

We asked participants about their demographic characteristics, including age, gender, education, and marital status. Also, we asked the participants about the perceived severity of COVID-19, perceived susceptibility to COVID-19, perceived self-efficacy in performing the COVID-19 vaccination and perceived response efficacy of COVID-19 vaccine, and intention to be vaccinated against COVID-19 whenever the vaccine was available. All answers were on 5-point Likert scales. We conducted this study in accordance with the Declaration of Helsinki, and the ethics committee of Zahedan University of Medical Sciences approved this study’s protocol (IR.ZAUMS.REC.1399.015).

### Data analysis

The analytical procedure consisted of two major tests: first, we performed confirmatory factor analysis (CFA). CFA examines the relationships between observed measures or indicators and latent variables or factors [21]. We checked the overall sample for the goodness of fit of the hypothetical measurement model of each domain, postulated by protection motivation theory developers. We performed structural equation modeling (SEM) to test for the proposed model in the next step. For investigating the fit of each model, we calculated the chi-square (χ2) statistic. However, this well-known statistic is not a useful model fit index practically because of the detection of even trivial differences under a large sample size [22]. Therefore, for more reliable results besides this test, we considered other goodness of fit indices like Comparative Fit Index (CFI), Tucker-Lewis Index (TLI), and Root Mean Square Error of Approximation (RMSEA) for a final decision about accepting or rejecting the hypothesis. A value of CFI ≥ 0.90, TLI ≥ 0.90, and RMSEA≤0.08 can support a good model fit [23]. We chose full information maximum likelihood estimation as estimators. CFA and SEM run by Mplus 8.3 [24].

## Results

### Participant characteristics

**Fig. 1:**
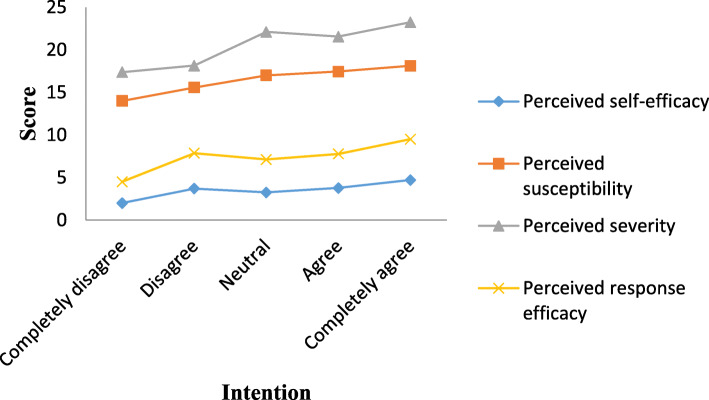
The survey responses in graphical form stratified by intent to get a vaccine

The average age of participants was 37.73 ± 12.27 years; 46.2% of them were male. 83.7% of participants had a university degree, 47.3% had an undergraduate degree, and 36.4% had a graduate degree. The survey responses in graphical form stratified by intent to get a vaccine are presented in Fig. 1.

We reported the descriptive statistics of measured variables in the model in Table 1, including skewness and kurtosis, which are indicators for univariate normality. The mean score range of items ranges from 3.208 to 4.475, and standard deviation scores range from 0.723 to 1.164. All items’ skewness and kurtosis scores fall in the acceptable ranges of normality suggested by Kline (skewness does not exceed |3| and kurtosis does not exceed |10|) [25].

**Table 1:** Descriptive statistics of the items in the measure

Construct and item	Mean	Standard deviation	Skewness	Kurtosis
Perceived susceptibility	17.478	1.164	−0.07	− 0.278
S6: I am at risk for COVID-19	3.671	1.047	−0.699	−.064
S7: I believe I am more likely to get COVID-19	3.208	1.102	0.008	−0.777
S8: The coronavirus will likely enter my body	3.295	1.101	−0.125	−0.791
S9: I am less at risk for COVID-19 than other members of my family	3.292	1.164	−0.251	−0.867
S10: If I contact a patient with COVID-19, my chances of getting COVID-19 are very high	4.004	0.870	−0.915	0.943
Perceived severity	22.045	3.841	−1.957	5.088
S1: I believe that COVID-19 is a serious problem	4.449	0.891	−2.144	4.962
S2: I believe that COVID-19 has bad effects on health	4.417	0.775	−1.863	4.982
S3: I believe that COVID-19 is a serious threat to my health	4.346	0.859	−1.568	2.437
S4: I believe that COVID-19 is a significant disease	4.458	0.723	−1.738	4.454
S5: I believe that COVID-19 can cause serious problems and even death	4.475	0.805	−2.002	4.655
Perceived self-efficacy: If a vaccine is produced, if I want, I’m sure I can get the COVID-19 vaccine	3.984	0.941	−0.9	0.783
Perceived response efficacy	8.220	3.751	−0.981	1.937
R1: If a vaccine is produced, vaccination reduces the risk of COVID-19 or its complications	4.064	0.827	−0.852	1.296
R2: If a vaccine is produced, vaccination will help me worry less about getting COVID-19	4.155	0.833	−1.212	2.194
Intention: If a vaccine is produced, I plan to get the COVID-19 vaccine	4.068	0.975	−1.161	1.457

We reported the Cronbach’s alphas, the composite reliability (CR), and the average variance extracted (AVE) in Table 2. All Cronbach’s alphas, CR and AVE, were greater than 0.70, indicating good reliability and validity of items within a construct (Table 2).

**Table 2:** Reliability analysis

	Cronbach’s alphas	Composite reliability	Average variance extracted
Perceived susceptibility	0.926	0.989	0.95
Perceived severity	0.772	0.986	0.944
Perceived response efficacy	0.848	0.969	0.941

CR for perceived severity and perceived response efficacy were 0.92 and 0.861, respectively, which were above the threshold of 0.7 AVE. Perceived severity and perceived response efficacy were 0.696 and 0.756, respectively, which were above 0.5. The discriminant validity results based on the Fornell-Larcker criterion are shown in Table 3.

**Table 3:** Discriminant validity

Constructs	Perceived severity	Perceived response efficacy
Perceived severity	0.696	
Perceived response efficacy	0.296	0.756

### Predictors of COVID-19 vaccination intention

**Fig. 2:**
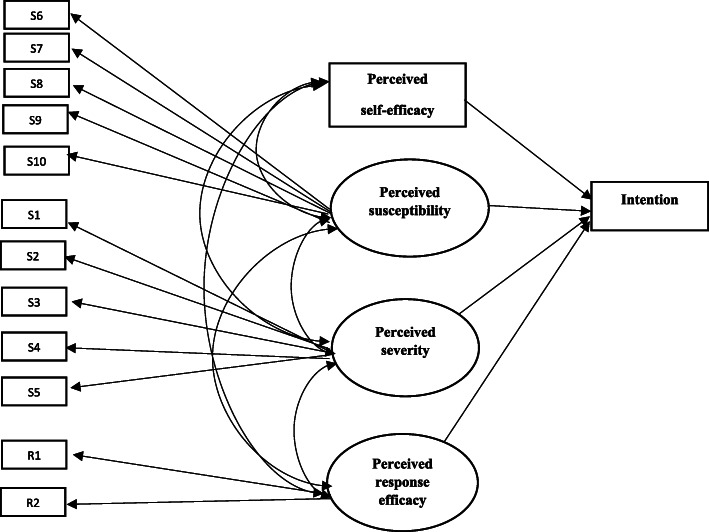
Perceived susceptibility, perceived severity, perceived self-efficacy, and perceived response efficacy were predictors of intention (Model1)

As mentioned earlier, the first step in testing SEM is to check whether the overall sample data fit the measurement model or not. The CFA analysis for all domains showed approximately acceptable CFI, TLI, and RMSEA values. Perceived susceptibility, perceived severity, perceived self-efficacy, and perceived response efficacy were predictors of intention in model 1. As shown in Table 4, the goodness of fit incidence of the model was *χ*^2^= 655.911, *P*-value< 0.001, CFI = 0.960, TLI = 0.950, and RMSEA =0.081. Although all goodness of fit indices were acceptable, perceived susceptibility was not significant, so we omitted perceived susceptibility to find a better model. Figure 2 also shows the graphical description of SEM analysis results. In Table 5, you can see all coefficients for the measurement model and path analysis.

**Table 4:** The goodness of fit index of models

	Chi-square	df	*P*-value	RMSEA	CFI	TLI
Measurement Model	160.062	51	< 0.001	0.091	0.941	0.923
Model 1	184.937	69	< 0.001	0.081	0.948	0.932
Model 2	77.343	23	< 0.001	0.096	0.966	0.947

**Table 5:** Standard estimation of model 1 parameters

		Estimate	S.E
Perceived susceptibility	S6	0.759	0.029
	S7	0.958	0.015
	S8	0.875	0.019
	S9	0.275	0.059
	S10	0.332	0.058
Perceived severity	S1	0.787	0.027
	S2	0.777	0.028
	S3	0.874	0.018
	S4	0.902	0.016
	S5	0.825	0.023
Perceived response efficacy	R1	0.834	0.026
	R2	0.904	0.022
Intention	Perceived susceptibility	0.028	0.045
	Perceived severity	0.119	0.048
	Perceived response efficacy	0.518	0.073
	Perceived self-efficacy	0.266	0.069
Perceived self-efficacy	Perceived response efficacy	0.75	0.032
	Perceived susceptibility	0.029	0.064
	Perceived severity	0.215	0.062
Perceived severity	Perceived susceptibility	0.328	0.061
Perceived response efficacy	Perceived susceptibility	0.087	0.068
	Perceived severity	0.296	0.065
Means	Perceived self-efficacy	4.131	0.192
Intercepts	S1	4.987	0.228
	S2	5.790	0.262
	S3	4.962	0.227
	S4	6.003	0.271
	S5	5.439	0.247
	S6	3.568	0.169
	S7	2.933	0.143
	S8	3.065	0.148
	S9	4.597	0.211
	S10	4.695	0.215
	R1	4.829	0.221
	R2	3.100	0.338
	Intention	2.841	0.139
variance	Perceived self-efficacy	1.000	0.000
	Perceived susceptibility	1.000	0.000
	Perceived severity	1.000	0.000
	Perceived response efficacy	1.000	0.000
Residual Variances	S1	0.381	0.043
	S2	0.396	0.043
	S3	0.236	0.032
	S4	0.187	0.028
	S5	0.319	0.038
	S6	0.424	0.044
	S7	0.082	0.029
	S8	0.234	0.033
	S9	0.890	0.038
	S10	0.305	0.043
	R1	0.182	0.040
	R2	0.385	0.041
	Intention	0.925	0.033

**Fig. 3:**
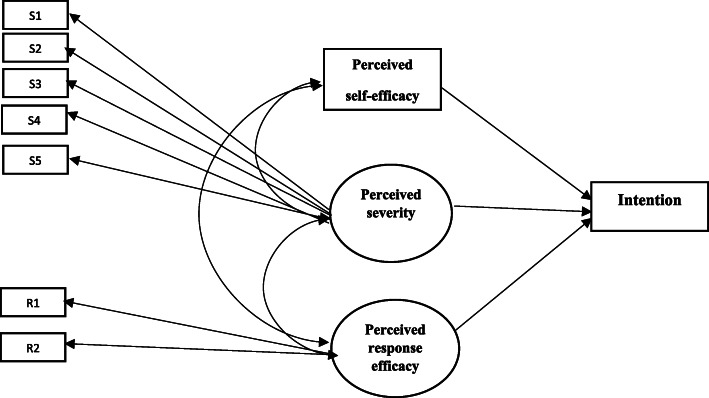
Perceived severity, perceived self-efficacy, and perceived response efficacy were predictors of intention (Model 2)

In model 2, perceived severity, perceived self-efficacy, and perceived response efficacy were predictors of intention. As shown in Table 4, the goodness of fit incidence of the model was *χ*^2^= 109.164, *P*-value< 0.001, CFI = 0.952, TLI = 0.933, and RMSEA =0.096. In this model, all goodness of fit indices are acceptable, and this model can explain 61.5% of the variance of intention. Figure 3 also shows the graphical description of the results of the SEM analysis. In Table 6, you can see all coefficients for the measurement model and path analysis. As shown in this Table, perceived severity to COVID-19 (β = .12, *p* < .001), perceived self-efficacy about receiving the COVID-19 vaccine (β = .26, p < .001), and the perceived response efficacy of the COVID-19 vaccine (β = .52, p < .001) were significant predictors of vaccination intention. Response efficacy was the strongest predictor of COVID-19 vaccination intention.

**Table 6:** Standard estimation of model 2 parameters

		Estimate	S.E
Perceived severity	S1	0.789	0.027
	S2	0.778	0.028
	S3	0.872	0.018
	S4	0.902	0.016
	S5	0.825	0.023
Perceived response efficacy	R1	0.834	0.026
	R2	0.904	0.022
Intention	Perceived severity	0.128	0.045
	Perceived response efficacy	0.520	0.073
	Perceived self-efficacy	0.263	0.069
Perceived self-efficacy	Perceived response efficacy	0.750	0.032
	Perceived severity	0.215	0.062
Perceived response efficacy	Perceived severity	0.296	0.065
Means	Perceived self-efficacy	4.131	0.192
Intercepts	S1	4.987	0.228
	S2	5.790	0.262
	S3	4.962	0.227
	S4	6.003	0.271
	S5	5.439	0.247
	R1	4.695	0.215
	R2	4.829	0.221
	Intention	3.110	0.337
variance	Perceived self-efficacy	1.000	0.000
	Perceived severity	1.000	0.000
	Perceived response efficacy	1.000	0.000
Residual Variances	S1	0.377	0.043
	S2	0.395	0.044
	S3	0.240	0.032
	S4	0.186	0.029
	S5	0.319	0.038
	R1	0.304	0.043
	R2	0.183	0.039
	Intention	0.386	0.041

## Discussion

Identification of factors influencing the acceptance of the COVID-19 vaccine should begin before a vaccine becomes available. The current study applies the PMT to identify predictors of COVID-19 vaccination intention in the Iranian adult population. We used SEM to investigate the interrelationship between COVID-19 vaccination intention and perceived susceptibility, perceived severity, perceived self-efficacy, and perceived response efficacy. The results showed that if the COVID-19 vaccine is available, the PMT could be a good predictor for vaccination intention. Previous studies that have used the PMT to predict vaccination intention have shown its effectiveness [26, 27]. A study that examined the predictor of seasonal influenza vaccination intention based on the PMT showed that the PMT accounted for 62% of vaccination intention variance [18].

The current study showed that perceived susceptibility to COVID-19 was not a significant predictor of vaccination intention. Participants in this study scored less than 70% of the maximum score of perceived susceptibility score, and this finding indicates that participants did not consider themselves very susceptible to COVID-19. In studies examining the intention to vaccinate against H1N1 influenza, perceived susceptibility to influenza H1N1 virus did not predict vaccination intention [28, 29]. Therefore, interventions should be designed and implemented by the health system to sensitize people to COVID-19. SEM showed that perceived severity to COVID-19, perceived self-efficacy about receiving the COVID-19 vaccine, and the perceived efficacy of the COVID-19 vaccine were significant predictors of vaccination intention. The three-factor model accounted for 61.5% of the total variance.

There is evidence that higher consideration of vaccination future consequences is associated with the perceived severity of the disease, greater perceived self-efficacy, and higher perceived effectiveness of the vaccine [30, 31]. An extensive survey that examined the willingness to vaccinate against seven vaccine-preventable diseases in the United States showed that different degrees of risk are associated with the number of people willing to be vaccinated [32].

Additionally, a study examining the acceptability of the COVID-19 vaccine found that participants who reported higher levels of perceived severity of COVID-19 infection and perceived effectiveness of COVID-19 vaccine were more likely to be willing to get vaccinated [5]. This study indicates that the perceived response efficacy is the strongest predictor of COVID-19 vaccination intention among the PMT construct. Regarding the effectiveness of the COVID-19 vaccine, other studies revealed that belief in vaccine efficacy was significantly the probability of COVID-19 vaccine acceptance [33, 34].

However, there is evidence that other factors can play a decisive role in influenza vaccination, despite understanding its effectiveness [35]. The previous research shows that perceived self-efficacy is one of the most critical factors in adherence to COVID-19 preventive measures [36]. Perceived self-efficacy refers to a sense of control over novel or difficult situations and challenges through decent behavior [37]. In behaviors such as vaccination that do not involve long-term treatment adherence, self-efficacy is a determinant of intention and behavior [38].

In a previous study that used PMT to predict staying at home during the COVID-19 pandemic in the Japanese population, self-efficacy was a predictor. Like this study’s results, perceived severity leads to threat appraisal more than perceived vulnerability, and perceived self-efficacy and perceived response efficiency leads to coping appraisal [39]. Also, evidence showed that perceived severity and self-efficacy were significantly related to the self-isolation intention during the COVID-19 pandemic [40].

Therefore, to encourage people to get vaccinated against COVID-19, more emphasis should be placed on perceived severity and perceived response efficiency. Because vaccination intention and actual vaccination uptake are related [41], identifying factors influencing vaccination intention before the availability of the COVID-19 vaccine can pave the way for community acceptance of the vaccine. Therefore, future intervention to increase COVID-19 vaccine acceptance can consider the PMT as a conceptual framework.

Readers should interpret our findings in light of the following study limitations. First, the COVID-19 vaccine is not yet available, and individuals’ answers to questions about vaccine efficacy and self-efficacy related to the vaccine may differ when the vaccine is available. Also, the distribution and cost of the vaccine are not known. If a vaccine provides in the future, the people who have access to the vaccine may have different characteristics from the participants in this study. Second, because we selected participants to study through an online survey platform, the findings may be prone to selection bias. Third, this study’s data were self-reported, and participants’ responses may prone to social desirability bias.

## Conclusions

The current study identified factors associated with the COVID-19 vaccination intention. Understanding the factors influencing vaccination can help health policymakers increase vaccine acceptance. Programs designed to increase the vaccination rate after the availability of the COVID-19 vaccine can include interventions on the severity of the COVID-19, the self-efficacy of individuals receiving the vaccine, and the effectiveness of the vaccine in preventing infection.

## Data Availability

The datasets used and analyzed during the current study are available from the corresponding author on reasonable request.

## Abbreviations

PMT: Protection Motivation Theory
SEM: Structural Equation Modeling
CFA: Confirmatory Factor Analysis
CFI: Comparative Fit Index
TLI: Tucker-Lewis Index
RMSEA: Root Mean Square Error of Approximation.
CR: Composite Reliability (CR)
AVE: Average Variance Extracted
